# Label free super resolution imaging with photonic nanojets from tunable tapered optical fibers

**DOI:** 10.1515/nanoph-2025-0404

**Published:** 2025-10-21

**Authors:** Maya Hen Shor Peled, Göran Maconi, Ivan Kassamakov, Alina Karabchevsky

**Affiliations:** School of Electrical and Computer Engineering, Ben Gurion University, Beer Sheva 8410501, Israel; Electronics Research Laboratory, Department of Physics, University of Helsinki, Helsinki, Finland; Department of Physics, Lancaster University, Lancaster LA1 4YB, UK

**Keywords:** photonic nanojet, super-resolution imaging, tapered fiber, light confinement, nano-optics

## Abstract

We demonstrate a label-free, far-field super-resolution imaging approach based on photonic nanojets generated by tapered dielectric fibers. By systematically analyzing the dependence of nanojet confinement and focal distance on cylinder diameter (8–16 μm), we establish a geometric design framework for tunable light localization below the diffraction limit. Using this insight, we fabricate a 12-μm waist-tapered optical fiber that produces a laterally extended nanojet for non-contact imaging. This configuration resolves grating lines with 92 nm width and spacing – dimensions beyond the classical resolution limit. Ray tracing simulations confirm the experimental magnification trend and show that fiber tilt enables tunable control over magnification and field of view. Our fiber platform provides scalable alignment, mechanical tunability, and extended working distances. These findings establish tapered fibers as compact and flexible photonic elements for delivering sub-wavelength light confinement, with applications in optical metrology, field enhancement, and scanning nanophotonic systems.

## 1 Introduction

Optical microscopy is a fundamental tool in scientific research, driving advancements in biomedicine, materials science, and nanotechnology. It remains the primary method for imaging biological samples by utilizing visible light and magnifying lenses [1], [2]. However, the wave nature of light imposes a fundamental resolution limit in conventional optical microscopy, first described by Ernst Abbe in the 19th century [3]. According to Abbe’s criterion, the spatial resolution of an optical system is limited to approximately half the wavelength of the illuminating light (*λ*). For violet light (*λ* = 400 nm), this results in a resolution limit of about 200 nm, which is sufficient for resolving cellular structures (1–100 μm) but inadequate for observing viruses, proteins and other nanoscale entities ranging from 1 to 100 nm.

Overcoming this diffraction limit is a major objective in optical imaging, as it enables molecular-scale investigations critical for understanding biological processes, developing disease diagnostics, and advancing nanoscale fabrication and inspection. The diffraction barrier arises under specific assumptions such as the use of a single objective lens, single-photon excitation and emission, and uniform illumination in the visible spectrum [4].

Many techniques have been developed to get around this limit. Near-field scanning optical microscopy (NSOM), electron microscopy (EM), and scanning probe microscopy (SPM) provide sub-diffraction resolution but involve trade-offs, including complex instrumentation, limited field of view, and the requirement for near-contact or vacuum environments. These methods are typically incompatible with live-cell imaging, soft materials, or dynamic *in situ* processes due to sample preparation constraints, vacuum requirements, and physical interaction with the specimen [5], [6], [7], [8]. Electron- and ion-based methods can also damage the sample or require coatings, limiting their use in many cases [9], [10].

Fluorescence-based super-resolution methods, such as stimulated emission depletion (STED), photoactivated localization microscopy (PALM), and stochastic optical reconstruction microscopy (STORM), have revolutionized optical microscopy by achieving resolutions well below the diffraction limit [11], [12], [13], [14], [15]. However, these methods are based on fluorescent labeling, which often involves complex sample preparation, potential impact on biological function, and susceptibility to photo-bleaching. They also require high-intensity excitation and long acquisition times, making them less suitable for live imaging of dynamic systems or for use on non-biological [16], [17].

Therefore, there is a growing interest in the development of label-free. These far-field super-resolution imaging methods preserve the integrity of the sample while maintaining compatibility with conventional optical setups. One such promising approach uses the phenomenon of photonic nanojets (PNJs) [18], [19], [20], [21]. A PNJ is a highly focused, non-evanescent light beam that forms on the shadow-side surface of a dielectric microstructure when illuminated by a plane wave. These beams exhibit sub-wavelength waist diameters and extended focal points, making them highly suitable for sub-diffraction-limit imaging applications. This phenomenon has been extensively studied for its potential in enhancing optical resolution beyond the diffraction limit [2], [18], [19], in different media and structure geometries [22], [23], as well as nano-patterning [24], signal enhancement for different characterization techniques [18], [25], [26] and nanoparticle manipulation [27], [28], [29], [30], [31], [32].

PNJs have been studied using microspheres to achieve high-resolution imaging [2], [33], [34], [35], but their practical use is often limited by a narrow field of view and the need for proximity to the sample. Typically, the field of view is confined to less than one-quarter of the microsphere’s diameter [36].

To overcome these limitations, studies have explored various strategies to enhance the field of view and flexibility of PNJ-based imaging systems. One approach involves the use of microsphere arrays to cover larger areas, although this introduces challenges in alignment and uniformity [37]. Another strategy employs scanning mechanisms to move a single microsphere across the sample surface, allowing the reconstruction of larger images from multiple frames [38], although this approach remains mechanically cumbersome and limited in lateral reach. These constraints motivate the exploration of cylindrical geometries, which naturally provide a more extended interaction region suitable for line-scanning applications. However, prior implementations of cylindrical PNJs often required physical contact with the sample, limiting their use in delicate or dynamic environments [39], [40].

In this work, we address these limitations by introducing a non-contact, tunable imaging platform based on a tapered optical fiber that generates a photonic nanojet. Compared to spherical geometries, the fiber’s cylindrical profile produces a laterally extended nanojet that offers a significantly larger field of view. By adjusting the fiber diameter and tilt, we can tune the focal point and magnification without changing the optical setup. These capabilities support dynamic imaging, flexible alignment, and straightforward integration into optical systems, making this approach well-suited for line-scanning metrology and compact super-resolution instrumentation.

We demonstrate the capability of this system by imaging sub-diffraction grating lines – 92 nm wide and spaced by 92 nm – using a 12 μm tapered fiber. To optimize the imaging performance, we first investigate the PNJ formation using both experimental characterization and simulations. We analyze how the full width at half maximum (FWHM) and focal point of the PNJ evolve with cylinder diameters ranging from 8 to 16 μm. Our results reveal that while the FWHM remains nearly constant, increasing only slightly with cylinder diameter (by less than 0.1 μm), the focal point extends nearly tenfold (by about 1 μm), offering enhanced flexibility in working distance without compromising on resolution.

By selecting an appropriate fiber diameter, we achieved sub-diffraction resolution while extending the working distance by up to 1 μm. This added distance allows non-contact imaging and reduces the risk of damaging delicate surfaces. In addition, the effective magnification can be tuned by adjusting the fiber tilt: maximum magnification is obtained when the fiber is parallel to the sample, and gradually decreases with angle. This controllable trade-off allows flexible adjustment between resolution and field of view, making the system well-suited for scanning-based imaging of nanoscale features.

## 2 Results and discussion

**Figure 1:**
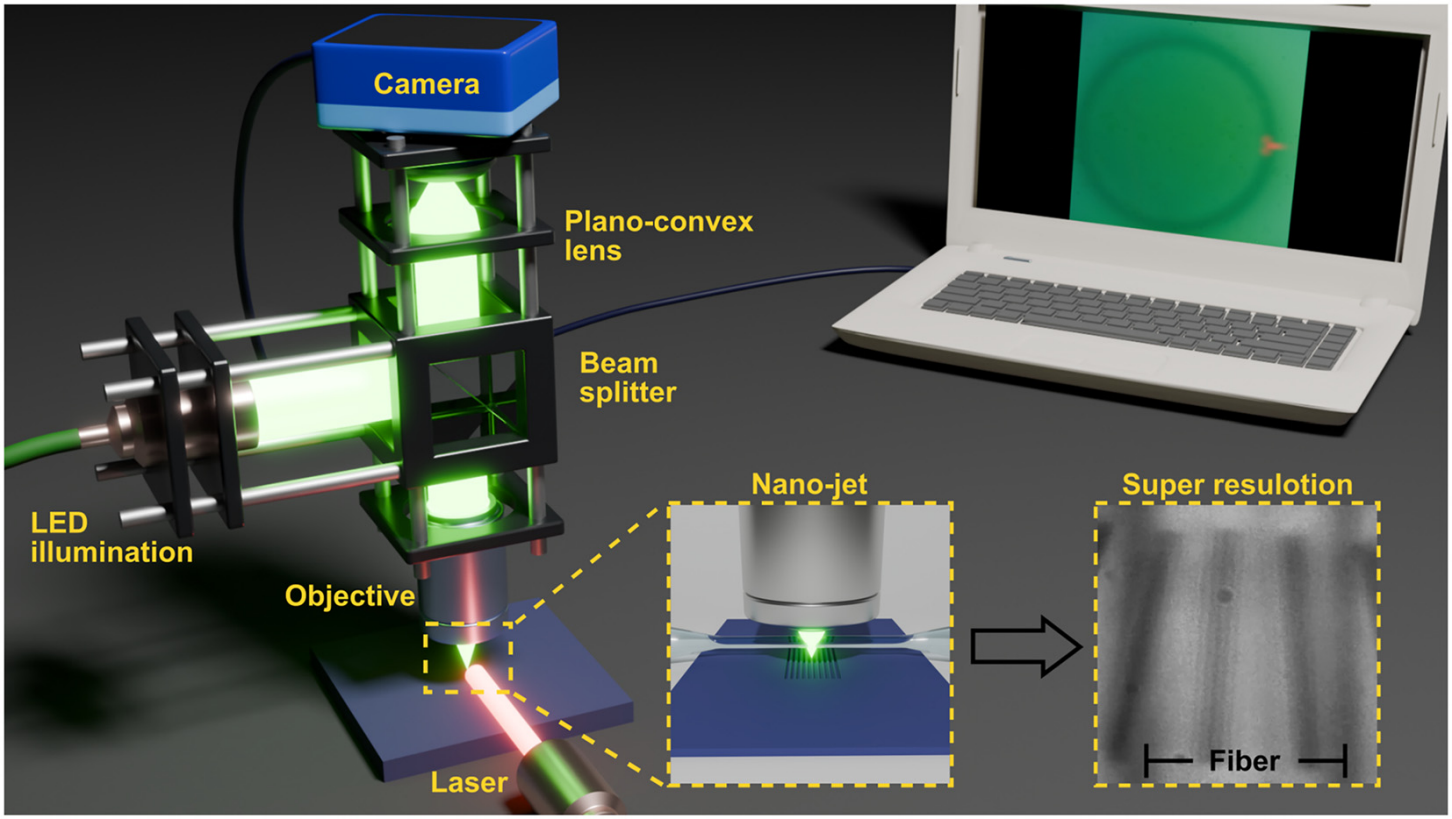
Illustration of the optical setup, as described in Section 4.2. A fiber-coupled green LED is collimated and passes through a 50/50 beam splitter, then directed onto the sample through an objective lens. The scattered light is collected by the same objective and directed to a plano-convex lens, focusing the light onto a CCD camera. To generate the photonic nanojet, a 637-nm laser illuminates the sample from the side through a collimator. The screen displays an experimentally captured image of a photonic nanojet generated by side illumination of a 16-μm diameter cylinder. The insets show a conceptual illustration of super-resolution imaging through the tapered fiber and an image of a 92-nm wide grating with 92-nm spacing captured by our system.

Figure 1 illustrates the experimental system, with subfigures highlighting the super-resolution imaging concept. A tapered fiber is illuminated from above while positioned over the sample of interest, in our case, a 92 nm grating with 92 nm spacing. The fiber generates a PNJ that interacts with the sample, and the scattered light is collected for imaging. To optimize the taper diameter, we first characterized PNJs formed by illuminating upright dielectric cylinders of various diameters from the side. Both imaging and PNJ characterization were performed using the same optical system.

**Figure 2:**
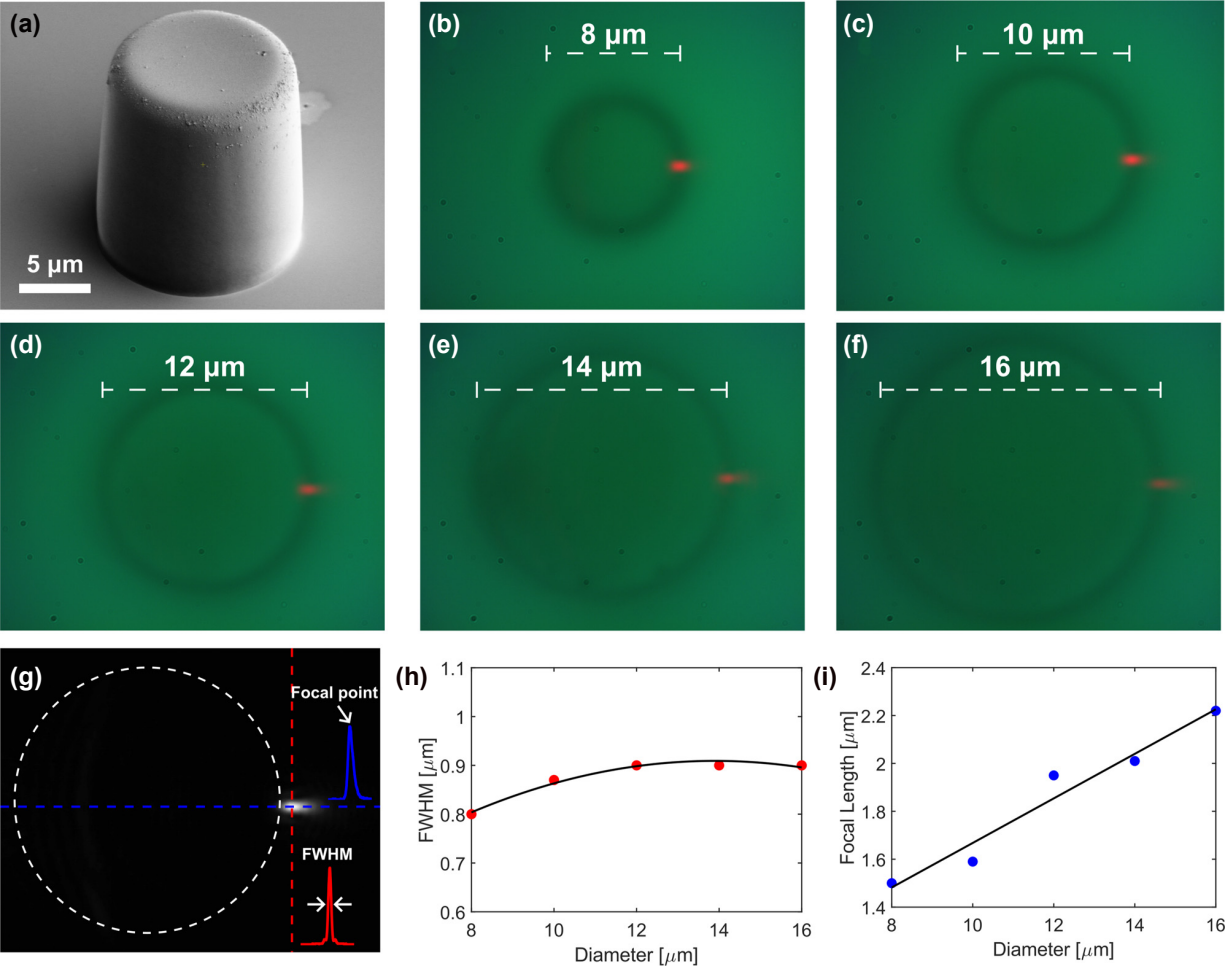
Experimental images of photonic nanojets generated by upright cylinders of different diameters. (a) SEM image of one of the five fabricated cylinders, with a diameter of 14 μm. (b)–(f) Show the photonic nanojets generated by side illumination of cylinders with diameters of 8, 10, 12, 14, and 16 μm, respectively. (g) Grayscale image of the 16 μm cylinder with vertical (red) and horizontal (blue) cross section lines placed at the point of maximal intensity. The insets show the corresponding intensity cross sections: red for vertical (used to calculate the FWHM) and blue for horizontal (used to calculate the focal point). (h) FWHM values as a function of cylinder diameter. (i) Focal point values as a function of cylinder diameter.

We fabricated five upright cylinders with diameters ranging from 8 to 16 μm (8, 10, 12, 14, and 16 μm) on a silicon substrate using the soft lithography technique with SU8 photoresist, as shown in Figure 2(a). This figure presents a scanning electron microscope (SEM) image of one of the fabricated pillars, with a diameter of 14 μm. The cylinders were illuminated from the side using a red laser (637 nm), and the resulting photonic nanojet images were captured with the optical setup described in Section 4.2.

Figure 2(b) to (f) display the experimentally captured images for cylinder diameters from 8 to 16 μm, respectively. To characterize the nanojets, we measured their full width at half maximum (FWHM) and focal point (the location of maximal intensity) by analyzing intensity cross sections along the vertical and horizontal directions at the point of maximal intensity, as illustrated in Figure 2(g). Figure 2(h) and (i) show the FWHM and focal point as a function of diameter. As the cylinder diameter increases, the focal point increases steadily from 1.5 to 2.2 μm. In contrast, the FWHM remains nearly constant, increasing slightly from 0.8 to 0.9 μm between 8 and 10 μm, and then saturating at 0.9 μm.

The measurement uncertainty is set by the CCD calibration (0.03 μm/px), corresponding to ±0.02 μm, while for simulations the finite mesh step (0.01 μm) gives an uncertainty of ±0.005 μm.

We aimed to compare the trends observed in our experimental results with theoretical predictions. To do so, we simulated the PNJ using Lumerical’s finite difference time domain (FDTD) method [41], calculating the electric field amplitudes generated by cylinders with diameters ranging from 8 to 16 μm. For each diameter, two simulations were performed: one with the electric field polarized along the *y* axis (TE) and another along the *z* axis (TM), both propagating along the *x* axis. Since our laser is polarized, we were particularly interested in assessing the effect of polarization on PNJ formation.

**Figure 3:**
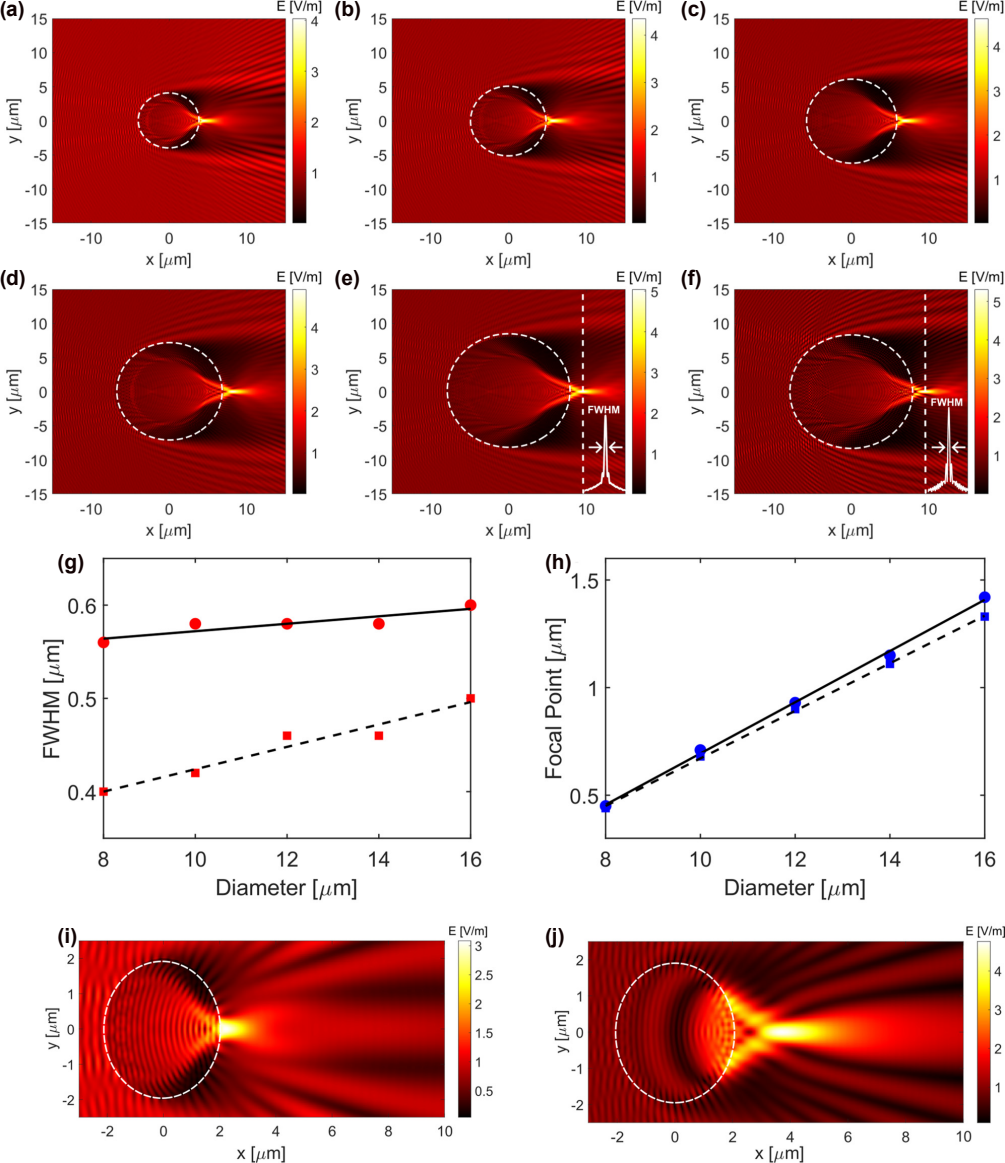
FDTD simulation of photonic nanojets generated by cylinders with varying diameters. (a) to (e) Show the electric field distribution for cylinders with diameters of 8, 10, 12, 14, and 16 μm, respectively, under TE polarization. (f) Shows the electric field for a 16 μm diameter cylinder under TM polarization. (g) and (h) Present the calculated full width at half maximum (FWHM) and focal point, respectively, as a function of diameter for TE simulation results (circles and solid line) and TM simulation results (squares and dashed line). (i) 2D FDTD simulation for a 4 µm diameter fiber segment. (j) Corresponding 3D FDTD simulation using the same refractive index and illumination.

Figure 3(a) to (e) show the electric field distributions under TE polarization for diameters from 8 to 16 μm, respectively, while Figure 3(f) shows the result for a 16 μm cylinder under TM polarization. The corresponding FWHM and focal point values for each configuration are summarized in Figure 3(g) and (h). The insets in Figure 3(e) and (f) show the intensity cross section used to calculate the FWHM.

Polarization influences photonic nanojet formation in both focal distance and lateral confinement. As seen in Figure 3(e) and (f) for a 16 μm cylinder, TE polarization (electric field along *y*) produces a nanojet that is broader laterally and extends farther, whereas TM polarization (electric field along *z*) yields a tighter (smaller FWHM) but shorter-focus nanojet. In our side-illumination experiments at 637 nm, the polarization-maintaining delivery and mount orientation resulted in TE polarization at the sample plane; accordingly, the experimental FWHM and focal-point trends align with the TE simulations in Figure 3(g) and (h). By contrast, the 505 nm LED used for grating imaging is unpolarized and low-coherence, so the effective imaging response averages over polarization states, with polarization-dependent interference effects strongly suppressed.

In the experimental results, the FWHM remains nearly constant, increasing slightly from 0.8 to 0.9 μm, while the focal point increases linearly from 1.5 to 2.22 μm. The TE simulation follows a similar trend, with FWHM ranging from 0.56 to 0.6 μm and focal point from 0.45 to 1.42 μm. In contrast, the TM simulation shows a consistently smaller FWHM, ranging from 0.4 to 0.5 μm, and a focal point trend similar to TE but with lower values across all diameters. The TE simulation trend aligns more closely with the experimental data, as expected given the TE polarization of the actual laser illumination in our setup.

Quantitatively, the experimental FWHM exceeds the TE simulation by about 0.25–0.3 μm, and the focal point is longer by roughly 0.9–1.0 μm. A key factor is the tapered geometry of the fabricated cylinders. SEM measurements show a base diameter of 14.34 μm and a top diameter of 12.97 μm, indicating a gradual change in width along the height. As our results demonstrate that the nanojet depends on cylinder diameter, this tapering likely contributes to the broader and more extended focal region observed in the experiment. Additional variation may result from differences in beam profile and intensity distribution, as well as finite resolution in image analysis, all of which can influence the extracted values. We additionally performed a finite-length 3D FDTD simulation for a 4 μm diameter fiber segment using the same refractive index and illumination as in our 2D case (Figure 3(i) and (j)). The 3D result exhibits a substantially longer focal length (1.69 μm vs. 0.22 μm) while the lateral FWHM remains similar, supporting our interpretation that tapering and finite axial extent of the fabricated structure lead to the observed discrepancies between measured and simulated PNJ parameters.

**Figure 4:**
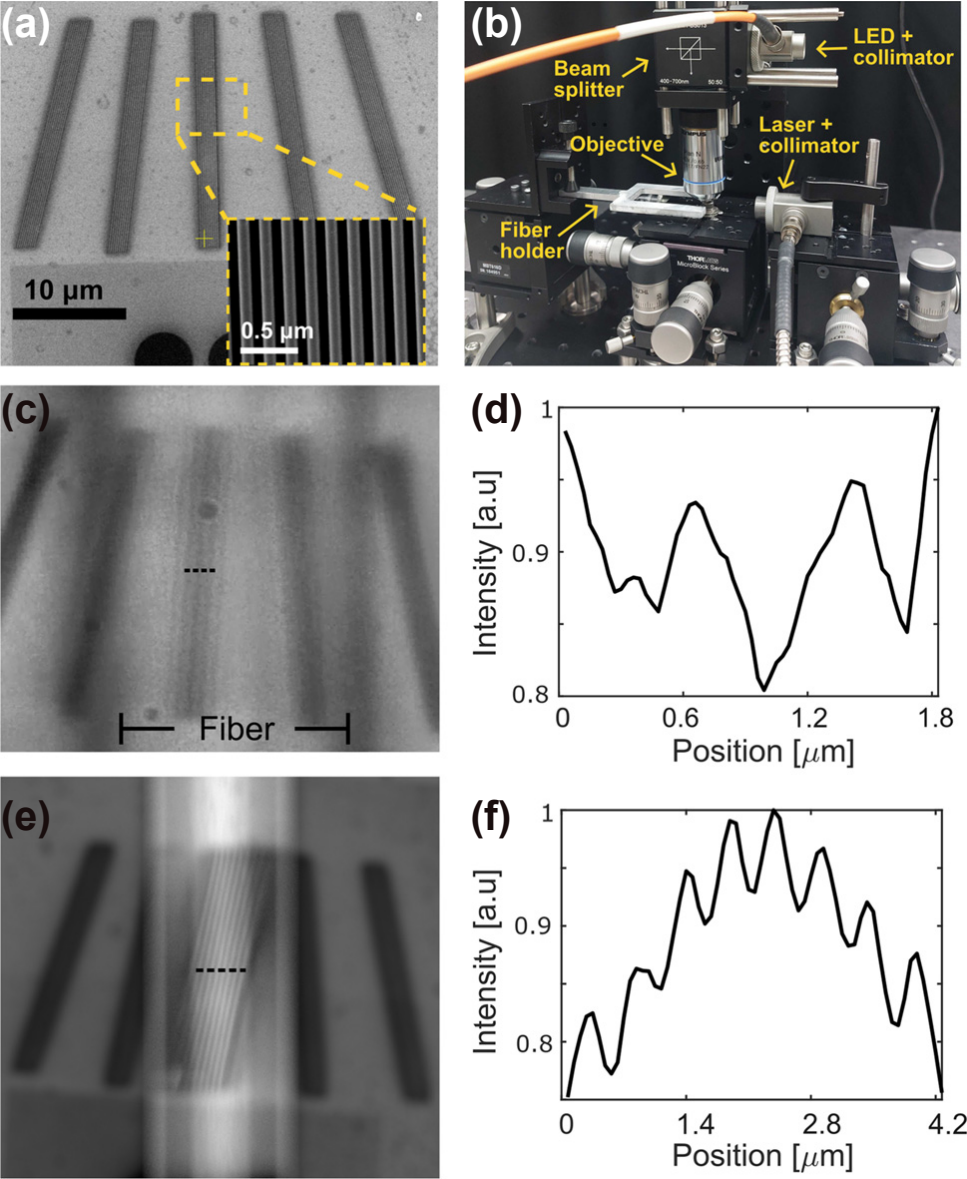
Super resolution imaging results. (a) SEM image of the grating sample, consisting of 10 lines approximately 92 nm wide with about 92 nm spacing, arranged in five angular configurations (−15°, −8°, 0°, 8°, and 15°); the inset shows a close-up of the middle grating. (b) Image of the optical setup, where the tapered fiber is held by a custom fork shaped holder mounted on a three axis translation stage. (c) Optical image of the middle grating captured through the tapered fiber, positioned directly above it. Only the grating lines aligned with the nanojet region are resolved, while the surrounding areas remain unresolved. (d) Intensity cross-section along the dotted line in (c). (e) Ray tracing simulation of the grating as imaged through the fiber. (f) Intensity cross-section along the dotted line in (e).

To utilize the photonic nanojet from the cylinder for super resolution imaging, we tapered an optical fiber from 125 μm to 12 μm to serve as the cylindrical structure. Figure 4(a) presents a scanning electron microscope (SEM) image of the fabricated grating sample, which consists of 10 lines approximately 92 nm wide with about 92 nm spacing, arranged in five angular orientations (−15°, −8°, 0°, 8°, and 15°). The inset shows a close up of the middle grating. Figure 4(b) shows the optical setup, where the fiber is held by a custom fork shaped holder mounted on a three axis translation stage. The fiber is positioned above the grating, and its height is finely adjusted along the *z* axis to optimize the nanojet focus. In contrast, the objective lens is focused beyond the grating plane. We note that the tapered optical fiber is not used to guide light internally. Instead, it serves as a movable cylindrical dielectric lens illuminated externally, allowing optimal positioning above the sample [18], [20], [21]. We emphasize that the illumination source is a broadband 505 nm LED with low temporal and spatial coherence. Unlike a coherent laser source, the LED strongly suppresses interference fringes and diffraction artifacts from the periodic grating.

In Figure 4(c), the tapered fiber is positioned directly above the middle grating, where the angle between the fiber axis and the grating lines is smallest. In this configuration, the fiber generated photonic nanojet enables clear visualization of the grating lines with 92 nm width and 92 nm spacing. These 92 nm features are more than a factor of four smaller than the Abbe diffraction limit (388 nm, *λ*/2NA with *λ* = 505 nm and NA = 0.65) of the objective lens, and therefore cannot be resolved without the subwavelength focusing provided by the photonic nanojet. We recognize that the term ‘super-resolution’ can refer to various physical or computational techniques. In this work, we define it strictly as achieving spatial resolution beyond the Abbe diffraction limit of the objective lens, without computational post-processing. Under this definition, our experimental results – resolving 92 nm wide and 92 nm spaced lines with a 0.65 NA objective – fulfil the super-resolution criterion. A real-time demonstration of the fiber scanning across the grating sample at different angles, with sub-diffraction lines visible without post-processing, is provided in Supplementary Video S1. In contrast, the surrounding gratings, which are not aligned with the fiber and lie outside the nanojet region, appear blurred and unresolved. This serves as an internal control within the same field of view, confirming that subwavelength features are only resolved when the tapered fiber generates a photonic nanojet above the sample. The intensity cross section along the dotted line in Figure 4(c) is shown in Figure 4(d), confirming the presence of subwavelength features in the magnified region.

To complement the experimental results and assess the imaging performance of the tapered fiber, we performed a ray tracing simulation using a Monte Carlo approach implemented in MATLAB. The fiber was modeled as a dielectric cylinder with a diameter of 16 μm, length of 220 μm, and refractive index of *n* = 1.47, consistent with the experimental conditions. A scaled scanning electron microscope (SEM) image of the grating was mapped onto a diffusely reflective planar surface to approximate realistic light scattering. Rays were traced in reverse from a virtual camera through the fiber and onto the object plane, simulating how light propagates through the system and forms an image.

Each pixel was sampled with 4,096 rays to ensure low noise and stable image reconstruction. The fiber was placed 20 nm above the reflective surface, avoiding physical contact while maintaining close proximity. The camera focus was set to a virtual plane located 15 μm beyond the object, reproducing the focal configuration used experimentally. Although the model does not account for wave optical effects such as diffraction, it provides a useful geometric approximation of the imaging behavior, capturing magnification, focal position, and field of view. The resulting simulated image is shown in Figure 4(e), with an intensity cross section plotted in Figure 4(f), closely matching the experimental image and confirming the fiber’s ability to resolve the subwavelength grating structure spatially.

To accurately quantify the magnification factor achieved by the tapered fiber, we analyzed both experimental and simulated images by measuring the periodic spacing between the grating lines. Specifically, we extracted the distances between successive intensity minima using line profiles along ten different cross sections in each image. These distances were initially recorded in pixels, then converted to physical units using the known pixel size and magnification of the imaging system.

**Figure 5:**
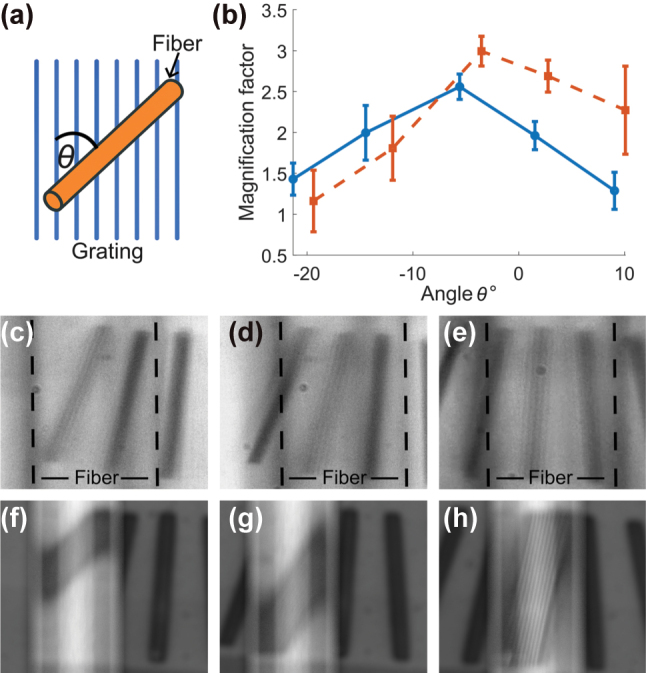
Magnification as a function of the angle between the fiber and the grating. (a) Illustration of the angle between the fiber axis and the grating lines. (b) Comparison of the calculated magnification factor as a function of this angle, showing both experimental results (blue circles with solid fit) and simulation results (orange squares with dashed fit). Error bars represent propagation from measurement variation across 10 sampling lines, uncertainty in SEM-measured grating pitch (5 nm), and estimated angle measurement error (±1°). (c) to (e) Experimental images of the fiber positioned over gratings at absolute grating angles of −15°, −8°, and 0°. (f) to (h) Corresponding simulation results for the same angles.

Because the fiber was positioned at a tilt relative to the grating sample, a geometric correction was applied to account for foreshortening. This correction was performed by dividing the measured feature sizes by cos(*θ*), where *θ* is the relative angle between the fiber axis and the grating lines. The maximum magnification was observed at a small but non-zero angle due to alignment tolerances, as also visible in Figure 5(a). The observed ∼5° offset reflects the manual placement of the fiber on the sample and is within the expected tolerance for such positioning. The angles used for correction were determined independently for the experimental and simulated cases, based on direct measurement from images, since the fiber alignment differed slightly between the two.

The magnification factor was then calculated as the ratio between the corrected feature sizes in the image and the known grating period (92 nm spacing, corresponding to 0.182 μm between dips). This procedure was applied to both experimental and simulated data. Errors were propagated from three sources: the standard deviation across the ten measurements, uncertainty in the SEM measurement of the grating spacing (estimated at 5 nm), and angle estimation error (±1°) based on manual alignment from image data.

The results are summarized in Figure 5(b). Experimentally, we observed a peak magnification of approximately 2.63 ± 0.15, with magnification decreasing as the fiber-grating angle increased. The simulation shows a similar trend, with a peak magnification of 2.94 ± 0.20, in good agreement with the experimental results. This inverse relationship between tilt angle and magnification illustrates how projection geometry affects the effective imaging performance of the tapered fiber. The more aligned the fiber is with the grating, the greater the projection length and magnification. This behavior highlights the role of angular alignment as a tunable parameter for optimizing resolution in nanojet-based imaging systems. Figure 5(a) illustrates the angle between the fiber and grating. Figure 5(c) to (e) show experimental images with three different angular orientations, and Figure 5(f) to (h) show the corresponding simulation results.

## 3 Conclusions

We demonstrated photonic nanojet-assisted super-resolution imaging with a tapered optical fiber as a label-free approach. To guide the fiber design, we first studied key parameters of photonic nanojets generated by upright dielectric cylinders of different diameters, illuminated from the side.

We found that increasing the cylinder diameter from 8 to 16 μm resulted in only a slight increase in the full width at half maximum (less than 0.1 μm), while the focal point increased by nearly a factor of ten. These trends closely matched simulations and confirmed that nanojet characteristics can be tuned geometrically to expand the working distance without compromising resolution.

Using these insights, we applied a tapered fiber with a 12 μm waist to image a nanoscale grating composed of 92 nm wide lines with 92 nm spacing. The experimental magnification reached 2.6, in close agreement with the simulated value of 2.9. As the tilt angle increased, the magnification decreased, consistent with projection geometry and enabling intuitive control over image scale and field of view. While this reduces image enlargement, it provides a simple way to adjust the field of view. This tunability, combined with the ability to image without contact, adds flexibility that is valuable in scanning applications or when imaging delicate surfaces.

This work establishes tapered fibers as compact and tunable imaging elements that combine mechanical simplicity with nanojet-based resolution enhancement. By integrating experiment, simulation, and geometric modeling, we demonstrated precise control over magnification, resolution, and focal distance. The strong agreement between measured and predicted behavior supports the use of this approach for scanning systems and non-invasive imaging of nanoscale structures. It offers a practical step toward accessible, label-free super-resolution imaging in compact and scanning-compatible platforms.

We note that the present study was performed on periodic grating samples with well-defined geometry. Extending this approach to random or curved nanoscale features, including biological matter, will be an important next step to demonstrate broader applicability. Future work will also explore integration of tapered-fiber nanojet imaging into optical metrology platforms, where compact, tunable, and label-free super-resolution could provide significant advantages for both biological and non-biological samples.

## 4 Experimental section

### 4.1 Fabrication

#### 4.1.1 Upright cylinder fabrication

Five upright cylindrical structures were fabricated using SU8 photoresist through a soft lithography process, following the method described in [42]. The resulting cylinders had diameters of 8, 10, 12, 14, and 16 μm, with a uniform height of 15 μm.

The fabrication began with a 2 × 2 cm silicon substrate (500 μm thick), which was first cleaned using a piranha solution (Hamatech) and then treated with a 5-min plasma ashing process (Diener electronic). GM1060 resist (Gersteltec Sarl., Switzerland) was spin-coated onto the substrate to achieve a layer thickness of approximately 15–16 μm. The spin-coating process included three stages: acceleration from 0 to 1500 rpm at 100 rpm/s for 15s, rotation at 1500 rpm for 40s, and deceleration back to 0 at 100 rpm/s for 15 s. The sample was then left to relax for 10 min.

A multi-step soft bake was applied by ramping the temperature from room temperature to 65 °C at 3 °C/min, holding at 65 °C for 10 min, then ramping to 95 °C at the same rate and holding for 35 min. The resist was exposed using an MLA150 system (375 nm wavelength) with a dose of 300 mJ/cm^2^ for 1 min. A post-exposure bake followed the same temperature profile, with a final hold at 95 °C for 30 min. After a 10-min rest, development was performed using AZ EBR for 1 min. A final hard bake was conducted at 135 °C for 2 h.

To remove any residual contaminants, the sample underwent an additional plasma ashing step for 3 min, with an etch rate of 100 nm/min. The fabricated structures were characterized using a confocal microscope (Olympus LEXT OLS5000) and a surface profilometer (DektakXT, Bruker).

#### 4.1.2 Tapered fiber

For the super resolution imaging experiments, we used a tapered optical fiber with a final waist diameter of approximately 12 μm to generate and direct photonic nanojets onto the sample surface. The fiber functioned as a compact dielectric cylindrical lens, producing localized light confinement beyond the classical diffraction limit and enabling label free imaging of nanoscale structures.

Tapering was performed using a precision glass processing system (Vytran GPX 3400, Thorlabs), which combines localized heating with automated mechanical pulling to produce smooth and reproducible fiber tapers. A standard single mode fiber with an initial cladding diameter of 125 μm was gradually reduced to 12 μm over a defined taper length. The tapering process was optimized to preserve adiabatic light guidance and minimize optical loss, while real time monitoring ensured geometric uniformity and structural continuity [43].

After fabrication, the tapered fiber was mounted on a custom three axis translation stage with submicron positioning accuracy. The fiber was aligned above the sample and brought into close proximity without physical contact. Fine adjustment along the vertical axis was used to optimize the nanojet focus within the imaging plane. This non-contact configuration allowed dynamic scanning and high resolution imaging of subwavelength grating features, establishing the tapered fiber as an effective and tunable nanojet delivery platform for far field super resolution microscopy.

#### 4.1.3 Grating

The nanoscale grating samples used in this study were fabricated using a dual-beam focused ion beam system (Helios G4 UC, Thermo Fisher Scientific), which integrates a high-resolution gallium ion column with a scanning electron microscope for *in situ* patterning and imaging. This system enables precise material removal with sub 10 nm resolution and is well suited for producing well defined and reproducible nanostructures with critical dimensions below 100 nm.

Each grating was patterned by etching parallel lines into a silicon substrate using a Ga^+^ ion beam operated at controlled energy and current to minimize collateral damage. The grooves were approximately 92 nm wide and spaced at a pitch of 92 nm, resulting in nearly equal width lines and gaps. The etch depth was tuned to approximately 100 nm to produce strong topographic contrast for photonic nanojet based imaging.

Each grating consisted of 10 lines, each approximately 15 μm in length, providing sufficient area for optical interrogation and enabling intensity profiling across multiple periods. To study the influence of angular alignment on magnification and resolution, five grating patterns were fabricated with orientations of −15°, −8°, 0°, 8°, and 15° relative to the horizontal axis. These angular variations were critical for evaluating the geometric sensitivity of the imaging setup and understanding the role of tilt in lateral resolution and magnification during both experimental and simulated imaging.

### 4.2 Experimental setup

The experimental setup, illustrated in Figure 1, was designed to support two complementary functions: (1) far field super resolution imaging using a tapered optical fiber, and (2) direct nanojet characterization using upright dielectric cylinders. This dual mode configuration allowed both the generation and evaluation of photonic nanojets as well as their application in imaging sub diffraction features.

The system was built around an epi illumination microscope platform. A fiber coupled LED source (505 nm, 8.5 mW; Thorlabs M505F3) provided broadband illumination, collimated by a reflective collimator (Thorlabs RC04FC P01) to form a uniform beam. The beam was directed through a 50:50 non polarizing beam splitter (Thorlabs CCM1 BS013), which transmitted part of the light toward the sample through a high numerical aperture objective lens. Two objectives were used interchangeably: a 100× objective (Zeiss LD EC Epiplan Neofluar 100×/0.75) for imaging the nanojets generated around dielectric cylinders, and a 40× objective (Olympus Plan N 40×/0.65) for capturing magnified images through the tapered fiber.

Reflected and transmitted light from the sample was collected by the same objective and directed back through the beam splitter toward the detection path. A plano convex lens (Thorlabs LA1484) focused the light onto a CMOS camera (Thorlabs DCC1645C), enabling high resolution image acquisition with submicron spatial sampling. This arrangement ensured minimal aberrations and high signal to noise for resolving nanojet features and magnified object details.

To generate the nanojet, a monochromatic 637 nm continuous wave laser (Qioptiq iFLEX iRIS CLM) was used for side illumination. The laser beam was fiber coupled and collimated with a second RC04FC P01 reflective collimator. This side illumination geometry was essential for producing well confined nanojets on the shadow side of the dielectric structures.

All optical elements, including the fiber, sample, objectives, and illumination sources, were mounted on *XYZ* translation stages (Thorlabs MBT616D) providing nanometer scale positioning. This allowed precise alignment of the fiber or cylinder relative to the sample surface, as well as fine control over focus. Although the laser is TM polarized by design and delivered via polarization maintaining fiber, its mounting orientation caused the illumination at the sample plane to be TE polarized.

Quantitative analysis of nanojet properties, including full width at half maximum and focal point, was performed using custom MATLAB scripts. The analysis involved extracting image cross sections, performing curve fitting, and calculating resolution metrics. This setup enabled direct comparison between simulation and experiment and validated the tapered fiber as an effective nanojet based imaging element.

### 4.3 FDTD simulation

The electric fields were simulated using the finite difference time domain method implemented in Ansys Lumerical FDTD [41], with perfectly matched layer boundary conditions applied at all edges. A total field scattered field source was used to launch a plane wave illumination. For each cylinder diameter, two simulations were performed: one with the electric field polarized along the *y* axis (transverse electric) and one along the *z* axis (transverse magnetic), both propagating in the *x* direction.

The illumination wavelength was set to 637 nm, matching the experimental laser. The cylinders were modeled in two dimensions with diameters from 8 to 16 μm and a refractive index of 1.6, consistent with SU-8 fabricated pillars. The surrounding medium was air. A uniform mesh with 0.01 μm resolution was applied along both *x* and *y* directions to ensure numerical accuracy. Field distributions were extracted using a frequency domain field and power monitor for quantitative analysis.

Post-processing of the simulation results was carried out in MATLAB. The exported electric field amplitudes were used to compute intensity maps, from which vertical and horizontal cross sections were extracted at the point of maximal intensity. The full width at half maximum was determined by identifying the lateral extent of the beam at half of its peak value. The focal length was defined as the distance from the cylinder boundary to the intensity maximum along the propagation direction. This approach enabled direct comparison with experimental measurements and supported the evaluation of nanojet focusing behavior across different cylinder geometries.

### 4.4 Ray-tracing simulation

To complement the experimental findings and assess the imaging performance of the tapered fiber, we performed numerical simulations using a Monte Carlo ray tracing approach. This method provides a geometric model of light propagation through the fiber, capturing key effects such as refraction, focusing behavior, and image magnification resulting from the fiber’s cylindrical shape.

The simulation traced 4,096 rays per pixel in reverse from a virtual camera through the fiber to the object plane, ensuring low noise for quantitative analysis. The object was defined using a scaled scanning electron microscope image of the actual grating, mapped onto a diffusely reflective planar surface to approximate realistic light scattering.

The imaging fiber was modeled as a dielectric cylinder with a diameter of 16 μm and length of 220 μm, with a refractive index of *n* = 1.47. It was positioned 20 nm above the reflective surface to match the experimental configuration while avoiding contact. The camera was focused at a virtual plane located 15 μm beyond the object to produce a sharp image through the fiber.

Although this model does not include wave optical effects such as diffraction, it offers a useful approximation of the imaging behavior and enables estimation of key parameters, including focal plane position, magnification, geometric aberrations, and field of view. The simulation results closely matched the experimentally measured magnification trends and provided further insight into the effects of fiber tilt on image scaling.
